# Expression of Bmi-1 is a prognostic marker in bladder cancer

**DOI:** 10.1186/1471-2407-9-61

**Published:** 2009-02-19

**Authors:** Zi-Ke Qin, Jian-An Yang, Yun-lin Ye, Xing Zhang, Li-Hua Xu, Fang-Jian Zhou, Hui Han, Zuo-Wei Liu, Li-Bing Song, Mu-Sheng Zeng

**Affiliations:** 1 State Key Laboratory of Oncology in South China, Sun Yat-sen University Cancer Center, Guangzhou, Guangdong 510060, PR China; 2 Department of Urology, Sun Yat-sen University Cancer Center, Guangzhou, Guangdong 510060, PR China; 3 Department of Experimental Research, Sun Yat-sen University Cancer Center, Guangzhou, Guangdong 510060, PR China; 4 Department of Biotherapy, Sun Yat-sen University Cancer Center, Guangzhou, Guangdong 510060, PR China

## Abstract

**Background:**

The molecular mechanisms of the development and progression of bladder cancer are poorly understood. The objective of this study was to analyze the expression of Bmi-1 protein and its clinical significance in human bladder cancer.

**Methods:**

We examined the expression of Bmi-1 mRNA and Bmi-1 protein by RT-PCR and Western blot, respectively in 14 paired bladder cancers and the adjacent normal tissues. The expression of Bmi-1 protein in 137 specimens of bladder cancer and 30 specimens of adjacent normal bladder tissue was determined by immunohistochemistry. Statistical analyses were applied to test the relationship between expression of Bmi-1, and clinicopathologic features and prognosis.

**Results:**

Expression of Bmi-1 mRNA and protein was higher in bladder cancers than in the adjacent normal tissues in 14 paired samples (*P *< 0.01). By immunohistochemical examination, five of 30 adjacent normal bladder specimens (16.7%) versus 75 of 137 bladder cancers (54.3%) showed Bmi-1 protein expression (*P *< 0.05). Bmi-1 protein expression was intense in 20.6%, 54.3%, and 78.8% of tumors of histopathological stages G1, G2, and G3, respectively (*P *< 0.05). Expression of Bmi-1 protein was greater in invasive bladder cancers than in superficial bladder cancers (81.5% versus 32.5%, *P *< 0.05). In invasive bladder cancers, the expression of Bmi-1 protein in progression-free cancers was similar to that of cancers that have progressed (80.0% versus 82.4%, *P *> 0.5). In superficial bladder cancers, the expression of Bmi-1 protein in recurrent cases was higher than in recurrence-free cases (62.5% versus 13.7%, *P *< 0.05). Bmi-1 expression was positively correlated with tumor classification and TNM stage (*P *< 0.05), but not with tumor number (*P *> 0.05). Five-year survival in the group with higher Bmi-1 expression was 50.8%, while it was 78.5% in the group with lower Bmi-1 expression (*P *< 0.05). Patients with higher Bmi-1 expression had shorter survival time, whereas patients with lower Bmi-1 expression had longer survival time (*P *< 0.05).

**Conclusion:**

Expression of Bmi-1 was greater in bladder cancers than in the adjacent normal tissues. The examination of Bmi-1 protein expression is potentially valuable in prognostic evaluation of bladder cancer.

## Background

Bladder cancer is one of the most lethal urological malignant tumors worldwide[1]. Although the treatment of bladder cancer has improved greatly in recent years, the incidence of this disease is gradually increasing [2]. More than half of the patients with bladder cancer have advanced stage disease with very poor prognosis[3]. Although some environmental factors and genetic factors have been associated with bladder cancer [4-6], the molecular mechanisms involved in the initiation and progression of bladder cancers remain unclear.

The *BMI1 *gene was first isolated as an oncogene that cooperated with c-Myc in generating lymphomas in a murine model[7,8]. It is a transcriptional repressor belonging to the Polycomb-group (PcG) family of proteins involved in axial patterning, hematopoiesis, regulation of proliferation, and senescence [9-11]. The *BMI1 *gene also was reported to immortalize bone marrow stromal cells and cementoblast progenitor cells, albeit in combination with other oncogenes [12,13]. In addition, the *BMI1 *gene plays an important role in cell proliferation and tumor progression [14-18]. The *BMI1 *gene is widely expressed in diverse human tumors, including lymphomas, non-small cell lung cancer, B-cell non-Hodgkin's lymphoma, breast cancer, colorectal cancer, and neuroblastoma [14-21], and has been shown to be a useful prognostic marker in myelodysplastic syndrome and many cancers, including nasopharyngeal carcinoma and gastric cancer [14-21].

However, there is no published report on the expression of Bmi-1 in bladder cancer. In this study we aimed to explore the expression of Bmi-1 protein and its clinical significance in human bladder cancers.

## Methods

### Patients and tissue specimens

For RT-PCR and Western blot analysis, we collected 14 paired transitional cell bladder cancers and adjacent normal tissues from the patients who underwent surgery between March 2008 and April 2008. In addition, 137 paraffin-embedded samples of transitional cell bladder cancer and 30 specimens of adjacent normal bladder tissue were collected between 2000 and 2003 for immunohistochemical assay. All tumors were histologically and clinically diagnosed by the cancer center of Sun Yat-sen University. For the use of these clinical materials, prior patient consent, and approval from the institute research ethics committee (Approval number: YP2008063) were obtained.

The disease stage of each patient was classified or reclassified according to the 2002 AJCC staging system[22]. The 137 patients included 115 males and 22 females from 14 to 72 years (mean, 56 years). Of these patients, 41 patients underwent radical cystectomy, 20 patients underwent partial cystectomy, and 76 patients underwent TURBT (transurethral resection of bladder tumor). After partial cystectomy and TURBT, mitomycin C was used in intravesical therapy as weekly intravesical injection beginning within 24 hours after surgery. Thirty specimens of adjacent normal bladder tissue distant from the tumor were included for these patients, as well. The median follow-up time for overall survival was 56 months for patients still alive at the time of analysis, ranging from 11 to 86 months.

### Reverse transcription – PCR analysis

Total mRNA was purified using TRIzol Reagent (Invitrogen, Carlsbad, CA, USA), and 1 μg of each sample was reverse transcribed using TIAN Script Kit (TIANGEN, Beijing, China). The *BMI1 *sense primer was 5'AGCAGAAATGCATCGAACAA-3', and the antisense primer was 5'CCTAACCAGATGAAGTTGCTGA-3'. For the β-actin gene, the sense primer was 5'CCCCTGGCCAAGGTCATCCATGACAACTTT-3', and the antisense primer was 5'GGCCATGAGGTCCACCACCCTGTTGCTGTA-3'. PCR reactions were performed following the cycling parameters on a PTC-200 PCR system (Bio-Rad, Hercules, Calif): 10 min at 94° followed by 28 cycles of 1 min at 94°, 1 min at 55°, 1 min at 72°, and a final cycle at 72°C for 10 min. PCR products were scanned, and quantification was performed by the Quantity One program (Bio-Rad, Hercules, CA).

### Western blot analysis

Total proteins were extracted with 1× SDS sample buffer [62.5 mmol/L Tris-HCl (pH 6.8), 2% SDS, 10% glycerol, and 5% 2-mercaptoethanol], and 30 μg of each protein was electrophoretically separated in 12% SDS polyacrylamide gels, and transferred to polyvinylidene difluoride membranes (Millipore). Rabbit polyclonal anti-Bmi-1 (1:800, Upstate Biotechnology, Lake Placid, NY) and anti-Rabbit (1:2000, Santa Cruz Biotechnology, Santa Cruz, CA) antibodies were used to detect Bmi-1 protein. Mouse anti-α-tubulin (1:2000, Sigma) and anti-mouse (1:2000, Santa Cruz Biotechnology, Santa Cruz, CA) antibodies were used to detect α-tubulin. The Western blot bands were scanned and were analyzed by the Quantity One program (Bio-Rad, Hercules, CA).

### Immunohistochemistry assay

Immunohistochemistry was performed to examine Bmi-1 expression in 137 human bladder cancers and 30 specimens of adjacent normal bladder tissue. The procedures were performed with classical protocols. In brief, paraffin-embedded specimens were cut into 4-μm sections and baked at 65° for 30 min. The sections were deparaffinized with xylene and rehydrated. Sections were submerged into EDTA antigenic retrieval buffer and microwaved for antigenic retrieval. The sections were then treated with 3% hydrogen peroxide in methanol to quench the endogenous peroxidase activity, followed by incubation with 1% bovine serum albumin to block the nonspecific binding.

The Bmi-1 protein was detected using a mouse monoclonal antibody against Bmi-1 (Upstate Biotechnology, Lake Placid, NY). The specimens were incubated with anti-Bmi-1 antibody (1:100) overnight at 4°. In the negative control, primary antibody was replaced by the non-immune mouse IgG of the same isotype. After washing, the tissue sections were treated with biotinylated anti-mouse secondary antibody followed by further incubation with a streptavidin-horseradish peroxidase complex. The tissue sections were immersed in 3-amino-9-ethyl carbazole, and counterstained with 10% Mayer's hematoxylin, dehydrated, and mounted in Crystal Mount.

The degree of immunostaining of formalin-fixed, paraffin-embedded sections was reviewed and scored by two independent observers. The proportion of cells expressing Bmi-1 varied from 0% to 100%, and the intensity of nuclear staining varied from weak to strong. Cells were scored for intensity of staining on a scale of 0 (no staining), 1 (weak staining, light yellow), 2 (moderate staining, yellowish brown), and 3 (strong staining, brown). Using this method of assessment, we evaluated the expression of Bmi-1 in bladder cancer tissue and in normal bladder tissue. An optimal cutoff value was identified. An intensity score of = 2 with at least 50% of malignant cells positive for Bmi-1 staining was used to classify tumors with high expression, and < 50% of malignant cells with nuclear staining of intensity score of < 2 characterized tumors with low expression of Bmi-1.

### Statistical analysis

All statistical analyses were carried out with the SPSS 13.0 statistical software package. In the RT-PCR and Western blot analysis, t-test was used to analyze the significance of mRNA and protein expression between bladder cancers and the adjacent normal tissues. We analyzed the relationship between expression of Bmi-1 protein, clinicopathologic features, and the clinical prognosis. The χ^2 ^test for proportion was used to analyze the relationship between Bmi-1 expression and clinicopathologic characteristics. Survival curves were plotted by the Kaplan-Meier method, and compared by the log-rank test. We determined that the assumption of proportional hazards was met in all Cox regression models. The significance of various variables for survival was analyzed by the Cox proportional hazards model in the multivariate analysis. *P *< 0.05 was considered statistically significant.

## Results

### Expression of Bmi-1 mRNA and protein in paired bladder tissues

**Figure 1:**
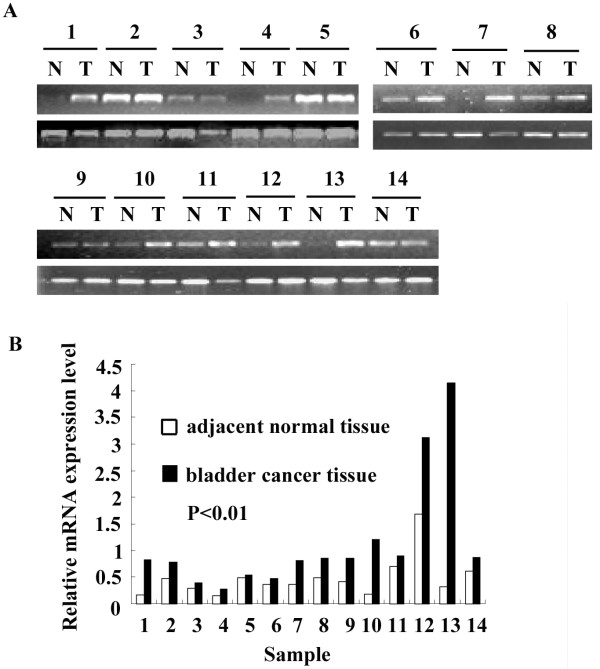
**Expression of Bmi-1 mRNA in paired bladder cancers and the adjacent normal tissues by RT-PCR analysis**. A: RT-PCR analysis in 14 paired bladder cancers and the adjacent normal tissues (upper panel); β-actin as the internal control (showed in lower panel). B: The relative expression level of Bmi-1 in comparison to the expression level of β-actin.

**Figure 2:**
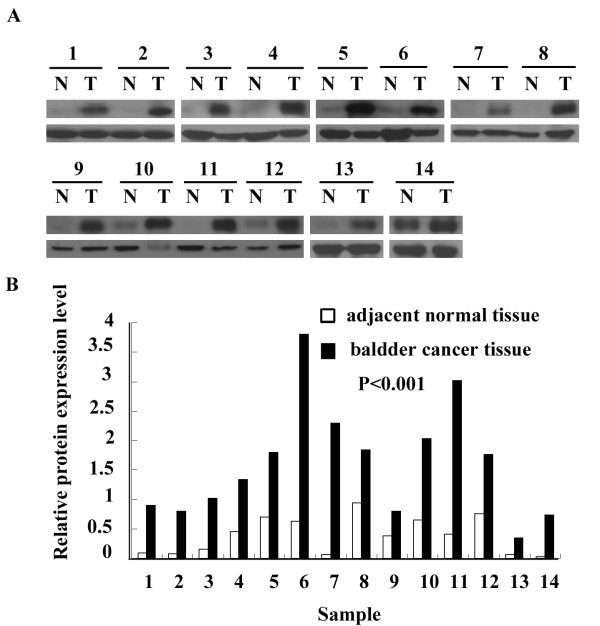
**Expression of Bmi-1 protein in paired bladder cancers and the adjacent normal tissues by Western Blot analysis**. A: Western Blot analysis in 14 paired bladder cancers and the adjacent normal tissues (upper panel); α-tubulin as the loading control (showed in lower panel). B: The relative expression level of Bmi-1 in comparison to the expression level of α-tubulin.

To ensure the reliability of this study, we first examined the expression of Bmi-1 mRNA and Bmi-1 protein by RT-PCR and Western blot, respectively in 14 bladder cancers. The control samples were paired adjacent normal tissues taken from the same patients. The relative level of Bmi-1 expression was compared in bladder cancers and the adjacent normal tissue (Figs. 1 and 2). The expression levels were determined as a ratio between Bmi-1 and the reference protein (α-tubulin) or gene (β-actin) to correct for variation in the quantity of protein and mRNA. In the RT-PCR analysis, the ratios (bladder cancer/normal tissue) were 4.97, 1.63, 1.37, 1.71, 1.09, 1.30, 2.19, 1.75, 2.05, 6.73, 1.28, 1.84, 12.67, and 1.42, respectively. In the Western blot analysis, the ratios (bladder cancer/normal tissue) were 10.71, 8.99, 6.05, 2.97, 2.55, 6.09, 35.47, 1.95, 2.12, 3.14, 7.42, 2.32, 5.46, and 32.47, respectively.

Expression of both mRNA and protein was found to be higher in tumor tissues than in the paired adjacent normal tissues (*P *< 0.01). Bmi-1 protein expression is up-regulated to a greater extent than is Bmi-1 mRNA expression in cancer tissues relative to non-cancerous tissues.

### Expression of Bmi-1 protein in paraffin-embedded bladder cancer samples

**Figure 3:**
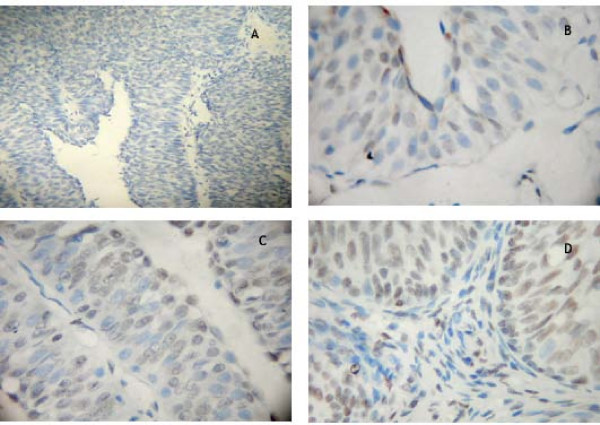
**Expression analysis of Bmi-1 protein by immunohistochemistry**. Bmi-1 staining was mainly localized within the nuclei, and it expressed was observed in cancer cell. A: Negative Bmi-1 staining in non-cancerous tissue (100×). B: Weak Bmi-1 staining in non-cancerous cells(400×). C: strong Bmi-1 staining in tumor nests(400×). D: Strong nuclear stainingin most of the tumor cells (400×).

Expression and subcellular localization of Bmi-1 protein were determined by immunohistochemistry in 137 paraffin-embedded bladder cancer tissues, and 30 specimens of adjacent normal bladder tissues. In non-cancerous tissues, Bmi-1 protein staining was present in few cells, and the protein staining was very weak (Fig. 3A, B). In tumor tissues, specific Bmi-1 signals were localized mainly in the nuclei of carcinoma cells in the form of yellow-brown granules (Fig. 3C, D). Bmi-1 protein expression was high in 54.3% of bladder cancers, while Bmi-1 expression was observed in only 16.7% of adjacent normal tissues (*P *< 0.05).

### Relationship between Bmi-1 protein expression and clinicopathological features

The relationship between the expression of Bmi-1 protein and clinical characteristics is shown in Table 1. Intense expression of Bmi-1 in bladder cancer was positively correlated with the histopathological classification, clinical stage, and recurrence (*P *< 0.05); but was not correlated with gender, age, or tumor number (*P *> 0.05). Intense expression of Bmi-1 protein was noted in 20.6%, 54.3%, and 78.8% of bladder tumors of histopathological grade G1, G2, and G3, respectively (*P *< 0.05, χ^2 ^test). Intense expression of Bmi-1 protein was noted in 81.5% of invasive bladder cancer and 32.5% of superficial bladder cancer (*P *< 0.05). In invasive bladder cancers, expression of Bmi-1 protein in progression-free cancers was similar to that of cancers with progression (80.0% versus 82.4%, *P *> 0.5). In superficial bladder cancers, Bmi-1 protein was highly expressed in 62.5% in recurrent cases versus 13.7% of recurrence-free cases (*P *< 0.05).

**Table 1:** Correlation between Bmi-1 expression and clinical-pathologic features of the patient with bladder cancer

		Bmi-1 expression		*P*
			
Characteristics	n	Low	High	
**Gender**				
Male	115	58 (50.4%)	57 (49.6%)	0.226
Female	22	8 (36.4%)	14 (63.6%)	
**Age**				
≤ 50	49	26 (53.1%)	23 (46.9%)	0.393
> 50	88	40 (45.5%)	48 (54.5%)	
**Tumor number**				
≤ 3	91	47 (51.6%)	44 (48.4%)	0.255
> 3	46	19 (41.3%)	27 (58.7%)	
**Histological grade**				
G_1_	34	27 (79.4%)	7 (20.6%)	0.001
G_2_	70	32 (45.7%)	38 (54.3%)	
G_3_	33	7 (21.2%)	26 (78.8%)	
**Clinical stage**				
T_a_, T_1_	83	56 (67.5%)	27 (32.5%)	0.001
T_2_, T_3_, T_4_	54	10 (18.5%)	44 (81.5%)	
**Superficial cancer**				
No recurrence	51	44 (86.3%)	7 (13.7%)	0.001
Recurrence	32	12 (37.5%)	20 (62.5%)	

### Survival analysis

**Figure 4:**
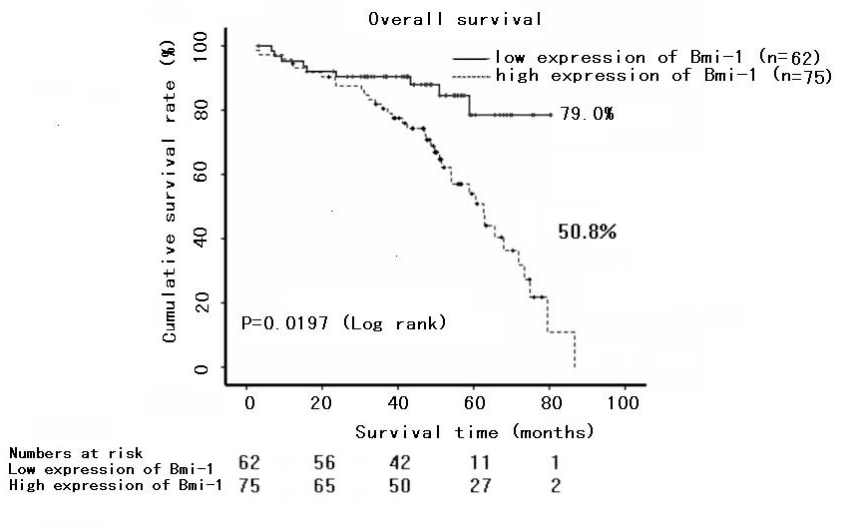
**Survival curves of patients with bladder cancer, subdivided according to Bmi-1 protein expression**. The 5-year survival rate was 79.0% in the Bmi-1(low) group (n = 62) and it was 50.8% in the Bmi-1(high) group (n = 75). The longest follow-up time is 86 months.

Kaplan-Meier analysis and the log-rank test were used to calculate the effect of the Bmi-1 expression on survival. The five-year survival in the group of higher Bmi-1 expression was 50.8%, but it was 79.0% in the group of lower Bmi-1 expression. The log-rank test showed that survival was significantly different between these two groups (*P *= 0.0197). The expression level of Bmi-1 protein in bladder cancer was significantly correlated with patient survival (*P *< 0.001), and the correlation coefficient was -0.27, indicating that a higher level of Bmi-1 expression was correlated with shorter survival time. The low Bmi-1 expression group had longer survival, whereas the high Bmi-1 expression group had shorter survival (Fig. 4). In addition, we did multivariate survival analysis, which included Bmi-1 expression level, histological classification, and clinical stage to test the independent effects of Bmi-1 on survival. We found that Bmi-1 expression, histological classification, and clinical stage were independent predictors of survival (Table 2). Thus, our findings indicate that the Bmi-1 protein expression level has a significant correlation with clinicopathological features, and is a potential prognostic marker for bladder cancer.

**Table 2:** Overall survival analysis of different prognostic variable in patients with bladder cancer by Cox Regression Analysis

		Univariate analysis		Multivariate analysis	
		
	n	*P*	Hazard ratios (95% confidence interval)	*P*	Hazard ratios (95% confidence interval)
**Clinical stage**					
T_a _T_1_	83				
T_2 _T_3 _T_4_	54				
**Histological grade**					
G_1_	34	0.003	2.412(1.302–3.673)	0.005	2.749 (1.437–3.758)
G_2_	70				
G_3_	33				
**Bmi-1 expression**					
High	75	0.022	1.653(1.121–3.485)	0.032	1.863(1.132–3.753)
Low	62				

## Discussion

At present, the molecular mechanisms of the initiation and progression of bladder cancers are unclear, although many genetic factors have been found to be associated with bladder cancer [4-6]. In this study, we report that Bmi-1 is overexpressed in human bladder cancers. The overexpression of Bmi-1 protein was correlated with tumor classification, recurrence, TNM stage, and prognosis. Patients with higher Bmi-1 expression had shorter survival time, and patients with lower Bmi-1 expression had a longer survival time.

This study demonstrated that there was a significant difference in Bmi-1 expression at both protein and mRNA levels between bladder cancer cells and the adjacent normal bladder tissue. Furthermore, Bmi-1 protein is up-regulated to a much greater extent than is Bmi-1 mRNA in cancer tissue compared with non-cancerous tissues. This observation suggests that dysregulation at the posttranscriptional level might be the major source of Bmi-1 expression in bladder cancers. Immunohistochemistry demonstrated that bladder cancers showed moderate to strong nuclear staining, while adjacent normal tissues showed only weak Bmi-1 expression, or no Bmi-1 expression at all. These results were similar to those of previous studies of other human cancers [14-18]. Furthermore, intense expression of Bmi-1 in bladder cancer correlated with its clinicopathologic features including tumor classification, recurrence, and TNM stage. Previous reports indicated that the *BMI1 *gene may be a novel molecular marker to predict the progression and prognosis of breast cancer and myelodysplastic syndrome (MDS)[19,20,23,24]. Consistent with previous reports of other cancers, over-expression of Bmi-1 protein indicated poor prognosis for patients with bladder cancer. The five-year survival was significantly different between the two groups, and the results showed that the greater the expression of Bmi-1 protein, the lower the survival rate.

The initiation and progression of bladder cancer involve a series of genetic events including activation of oncogenes, and the inactivation of tumor suppressors[6,25,26]. The regulatory mechanism of the Polycomb group proteins relies upon epigenetic modifications of specific histone tails that are inherited through cell division[10,27]. Because Bmi-1 is a member of the PcG family, Bmi-1 overexpression could repress the p16Ink4a (*CDKN2A*) and p19Arf targets[17,20]. In the absence of p16Ink4a, the cyclin D/Cdk4/6 complex can phosphorylate pRB, allowing the E2F-dependent transcription that leads to cell cycle progression and DNA synthesis. Bmi-1-deficient mouse embryonic fibroblasts (MEF) overexpress INK4a/ARF locus-encoded genes *CDKN2A *and p19ARF (mouse homologue of human p14ARF) and undergo premature senescence in culture[28].

Conversely, overexpression of Bmi-1 reduces expression of p16INK4a and p19ARF, and immortalizes MEFs[28]. Kang[29] found that Bmi-1 may act through a p16INK4A-independent pathways to regulate cellular proliferation during progression of oral cancer. In addition, MDM2-mediated p53 degradation causes low p53 levels in the absence of p19Arf, thus preventing cell cycle arrest and apoptosis[30]. The *BMI1 *gene also can induce telomerase activity to prevent apoptosis. Other investigators have found that the frequent inactivation of the p14ARF and *CDKN2A *genes may be an important mechanism for the dysfunction of p53 and Rb growth regulatory pathways during bladder cancer development [31-34]. However, whether overexpression of *BMI1 *results in reduction of p14ARF and *CDKN2A *gene expression requires further investigation.

In our experience, the clinicopathologic features of bladder cancer are closely related to its prognosis. In our study, we found that in addition to T classification and TNM stage, Bmi-1 expression is an independent prognostic factor for bladder cancers. In the literature, the *BMI1 *gene is reported to be related to the clinicopathologic features and prognosis in other human cancers, including breast cancer, colon cancer, and lung cancer [19-21]. Thus, we believe that the *BMI1 *gene probably plays an important role in cell proliferation and tumor progression in bladder cancers. More studies are required to explore the relationships between the *BMI1 *gene and other genes such as p14, p16, and *TP53*, and its relationship to other molecules that may be associated with bladder cancer.

## Conclusion

This study demonstrated overexpression of Bmi-1 in bladder cancers. The overexpression of Bmi-1 protein was correlated with tumor classification, recurrence, clinical stage, and prognosis. Greater expression of Bmi-1 protein predicts a lower degree of differentiation, higher recurrence risk, and poorer prognosis. These results indicate that the *BMI1 *gene may play an important role in the progression of bladder cancer; however, this study has several limitations. In this study, the old WHO grading system (grades 1, 2, and 3) was used, and lymphovascular invasion was not recorded. In addition, we can say only that Bmi-1 expression is an independent prognostic marker for bladder cancer. It may offer new information that stage and grade cannot provide, but more evidence is required to support this conclusion. Prospective studies, additional cases, and different antibodies would be required to test the relationship between Bmi-1 expression and the clinical biological behavior of bladder cancer.

## Competing interests

The authors declare that they have no competing interests.

## Authors' contributions

ZKQ, JAY and YLY were responsible for data collection and analysis, experiment job, interpretation of the results, and writing the manuscript. FJZ, HH, XZ, ZWL and SLB were responsible for conducting the data analysis, reviewing and scoring the degree of immunostaining of sections in cooperation with JAY. MSZ and ZKQ were responsible for experimental design, analysis and interpretation. All authors have read and approved the final manuscript.

## Pre-publication history

The pre-publication history for this paper can be accessed here:

http://www.biomedcentral.com/1471-2407/9/61/prepub
